# Fragility of Randomized Clinical Trials Using Small Bite, Small Walk Versus Large Bite, Large Walk in Laparotomy Closure

**DOI:** 10.1097/AS9.0000000000000640

**Published:** 2026-01-08

**Authors:** Lucy R. Hinton, Samantha W. Kerr, Victoria L. Walker, Gregory T. Scarola, Sullivan A. Ayuso, B. Todd Heniford

**Affiliations:** From the * Division of Gastrointestinal and General Surgery, Department of Surgery, Endeavor Health, Evanston, IL; † Division of Gastrointestinal and Minimally Invasive Surgery, Department of Surgery, Carolinas Medical Center, Charlotte, NC; ‡ Department of Surgery, Baptist Health Sciences University, Memphis, TN; § Division of Elective General Surgery, Department of Surgery, University of Texas, Austin, TX.

## INTRODUCTION

The Fragility Index (FI) was designed to assess the robustness of outcomes of randomized controlled trials (RCTs) and complement standard statistical analysis, such as the *P* value, by reflecting the robustness of the study.^1^ It represents the minimum number of participants in RCT whose outcome status would need to change to turn a statistically significant result into a nonsignificant result (Table 1). A Reverse Fragility Index (RFI) is the number needed to change a nonsignificant result to significance.^2^ A FI ≤ 3 is generally considered fragile.^1^ Despite the categorical FI, if the number lost to follow-up exceeds the FI, the robustness of the outcome is diminished, as the missing data could reverse the study’s findings. Fragility quotient and reverse fragility quotient evaluate the FI/RFI relative to the sample size.^1^ FI has been used to assess the robustness of the use of mesh in Abdominal Wall Reconstruction.^3^ The optimal fascial closure technique after laparotomy to decrease incisional hernia formation has been closely studied.^4^ Updated guidelines from the European and American Hernia Societies recommend a continuous small-bites suturing technique.^5^ Given that fragility analysis may influence how such recommendations are interpreted, the aim of this study was to review the fragility of RCTs that assess small bite, small walk versus large bite, large walk in laparotomy closure.

**TABLE 1.** How to Calculate the Fragility Index (FI)

Trial Result			Calculated Fragility		
	Event	No Event		Event	No Event
Treatment A	a	b	Treatment A	a + f	b − f
Treatment B	c	d	Treatment B	c	d
Fisher Exact Test *P* < 0.05			Fisher Exact Test *P* ≥ 0.05		

## METHODS

A systematic search of PubMed and Google Scholar was performed for studies between January 2000 and June 2025 with the search terms: “Large OR small OR long OR short”, “suture OR sutures OR stitch”, “midline incision OR median laparotomy”, “randomized controlled trial”. The first 200 studies from each search engine were reviewed, as this was deemed to be the theoretical data saturation point after which no new information was being found. RCTs comparing small bites (bites of 5–8 mm and intersuture spacing of 5 mm) with large bites (bites of at least 10 mm and intersuture spacing of 10 mm) for closure of the abdominal fascia after elective or emergency midline laparotomy with prevention of incisional hernia as its primary outcome. A total of 6 RCTs were included. *P* values were recalculated with a Fisher exact test if a χ^2^ test was used, significance was set at *P* < 0.05. Data were analyzed in July 2025.

## RESULTS

A total of 6 RCTs were included: 4 (66.7%) reported statistically significant results favoring small-bite, small-walk, while 2 (33.3%) were negative. The median (interquartile ratio) FI for positive RCTs was 5.50 (1.50–12.0), and the median (range) RFI for negative RCTs was 2.00 (2.00–2.00). Two (50.0%) of the 4 positive studies had a FI of 3 or less, indicating a less robust study. The median (interquartile ratio) fragility quotient was 0.018 (0.003–0.038), and the median reverse fragility quotient (range) was 0.012 (0.005–0.020). One of the positive studies was no longer statistically significant when a Fisher exact test was used to calculate the *P* value (*P* = 0.083), resulting in a FI of 0. In the trial with the largest FI (FI = 16), the loss to follow-up (n = 147; 19.9%) far exceeded the FI, introducing potential fragility, as outcomes of these missing participants could undermine the study conclusions.

## DISCUSSION

The findings of this study show that FI is a useful adjunct when assessing the outcomes of statistically significant studies. Despite statistical significance, a fragile result may not be clinically durable and should be considered before practice-changing guidelines. Across the 6 RCTs evaluating the small-bite, small-walk technique for midline laparotomy closure, results have been heterogeneous. Two of the 4 (50.0%) positive studies were found to be quite fragile when calculating the FI, including one that lost statistical significance when the Fisher exact test was used. The study with the most robust FI had a loss to follow-up of >9 times the FI, introducing uncertainty as the unobserved outcomes could feasibly reverse the apparent significance of the findings. A limitation of FI/RFI is that it applies to only binary outcomes, and, therefore, is unable to be used for continuous outcomes, which are often valuable metrics when assessing surgical outcomes, including operative time, cost, and quality of life.

Ultimately, the fragility of the evidence base should temper the strength of current recommendations favoring small-bite, small-walk closure.

## References

[1] Tignanelli CJ Napolitano LM. The fragility index in randomized clinical trials as a means of optimizing patient care. JAMA Surg. 2019;154:74–79. 30422256 10.1001/jamasurg.2018.4318

[2] Khan MS Fonarow GC Friede T. Application of the reverse fragility index to statistically nonsignificant randomized clinical trial results. JAMA Netw Open. 2020;3:e2012469. 32756927 10.1001/jamanetworkopen.2020.12469 PMC7407075

[3] Ayuso SA Holland AM Lorenz WR. Fragility of randomized clinical trials using mesh in abdominal wall reconstruction. JAMA Netw Open. 2023;6:e2347534. 38091044 10.1001/jamanetworkopen.2023.47534 PMC10719754

[4] Henriksen NA Deerenberg EB Venclauskas L. Meta-analysis on materials and techniques for laparotomy closure: the MATCH review. World J Surg. 2022;46:848–858. 10.1007/s00268-017-4393-9 29322212

[5] Deerenberg EB Henriksen NA Antoniou GA. Updated guideline for closure of abdominal wall incisions from the European and American Hernia Societies. Br J Surg. 2022;109:1239–1250. 36026550 10.1093/bjs/znac302 PMC10364727

